# Similarities and Differences in Health, Social Trust, and Financial Situation in People With Usher Syndrome, a Bio-Psychosocial Perspective

**DOI:** 10.3389/fpsyg.2020.01760

**Published:** 2020-08-28

**Authors:** Moa Wahlqvist, Claes Möller, Kerstin Möller, Berth Danermark

**Affiliations:** ^1^Audiological Research Center, Faculty of Medicine and Health, Örebro University, Örebro, Sweden; ^2^Swedish Institute for Disability Research, wÖrebro University, Örebro, Sweden; ^3^The Swedish National Resource Center for Deafblindness, Lund, Sweden

**Keywords:** bio-psychosocial perspective, deafblindness, financial situation, health, physical health, psychological health, social trust, Usher syndrome

## Abstract

**Purpose:**

The primary aim was to describe the similarities and differences among the general health, physical health, psychological health, social trust, and financial situations of people with Usher syndrome (USH) types 1, 2, and 3. A second aim was to explore whether age, gender, clinical diagnosis, visual field, visual acuity, and degree of hearing impairment were associated with the general health, physical health, psychological health, social trust, and financial situations of people with USH.

**Methods:**

In this study, 162 people with USH living in Sweden were included, and all three types of the disease were represented. Data concerning vision, hearing, and genetics were retrieved from the Swedish Usher database. Group comparison using frequencies, χ^2^-tests and Kruskal-Wallis tests for group comparison were used. To examine the effect of independent variables on poor health outcomes, a logistic regression analysis was conducted.

**Results:**

Problems with poor health, social trust, and finances were found for all three types; however, more similarities than differences were found. The results of the regression model were ambiguous; it is not clear which independent measures contributed the most to poor outcomes. People with USH3 tended to report the most problems regarding the dependent outcome measures.

**Conclusion:**

The observations of the associations between the independent variables and poor health, social trust and finances made in the present study are important to bear in mind in a rehabilitation setting; however, they do not fully explain how people with USH actually feel or rate their health. More research is needed to confirm the knowledge that exists within the clinical setting and the life stories told by the people with USH to merge existing knowledge into a rehabilitation setting based on evidence.

## Introduction

People with disabilities are more vulnerable to poor health status and comorbidities (Verbrugge et al., 1989; Abdullah et al., 2004). However, conducting research within this area is challenging because of the extant diversity among people with different disabilities and within groups of people with the same conditions. People with deafblindness [i.e., those with combined visual and hearing impairment (HI)] are a vulnerable group in society because their impairment severely restricts information, interactions, and communication with others (World Federation of the Deafblind, 2018). Because vision and hearing are complementary senses and enhance each other, impairments in one sense (i.e., hearing or vision) make it possible to compensate for restrictions in the other. However, for those with deafblindness, the ability to compensate becomes restricted due to impairments in both senses (Möller C., 2003). As defined in the Nordic definition of deafblindness: *“Deafblindness is a combined vision and hearing impairment of such severity that it is hard for the impaired senses to compensate for each other. Thus, deafblindness is a distinct disability. To varying degrees, deafblindness limits activities and restricts full participation in society”* (Nordic Centre for Welfare and Social Issues, 2016).

The most common cause of genetic deafblindness is Usher syndrome (USH; Kimberling and Möller, 1995). To date, 13 genes have been identified causing USH (Mathur and Yang, 2015). Three clinical types of USH are known (USH1, USH2, and USH3), and they differ clinically in their severity and the onsets of hearing and vision impairments. For some, the vestibular organ is affected, with congenital bilateral vestibular areflexia and balance problems (Kimberling and Möller, 1995). People with USH1 are characterized as having congenital, severe- to- profound HI, bilateral vestibular areflexia and the onset of retinitis pigmentosa (RP) during the first decade of life. Retinitis pigmentosa is a progressive eye disorder that affects the rods and cones in the retina. Early symptoms are night blindness, a narrowing visual field, additional light and contrast sensitivity, and impaired visual acuity. Due to vestibular areflexia, people with USH1 show a delayed walking age (>18 months). Cataract often accompanies RP. USH2 is characterized by a moderate- to-severe congenital HI and a late diagnosis of RP during the second decade of life. However, visual problems might be present earlier. People with USH3 experience progressive hearing, vision and balance loss. Thus, the HI suffered during childhood is moderate but progresses to severe or profound in adulthood. For people with USH3, the vestibular balance function can progressively degenerate. Both the clinical and heterogeneity of this condition is vast, and the genes interact in networks (Millán et al., 2011; Bonnet and El-Amraoui, 2012; Mathur and Yang, 2015). People with USH use different ways to communicate with others (e.g., speech, lip-reading, visual sign language, and tactile sign language) as well as to receive and provide information. They also use different types of support from others (e.g., interpreters or guides) and, technical devices (e.g., hearing aids, cochlear implants, loop systems, computers, magnifiers, braille script readers); moreover, it is common for communication strategies to change throughout life (Ellis and Hodges, 2013).

Previous research has shown that people with deafblindness are vulnerable to different mental health conditions, such as depression and anxiety (Miner, 1995, 1997; Bodsworth et al., 2011) as well as uncertainty of what the future will bring when living with a disease with a progressive course (Fletcher and Guthrie, 2013). The study by Bodsworth et al. (2011) did not address people with USH exclusively but focused only on adults with deafblindness who were members of Deafblind UK and responded to a questionnaire concerning psychological health as well as informal and formal support. In two studies, Miner asked people with USH1 or USH2 to describe living with deafblindness, the challenges faced and adaptations made, from a life course perspective (Miner, 1995, 1997). Social withdrawal and isolation are also consequences of deafblindness (Bodsworth et al., 2011; Hersh, 2013). The consequences of living with deafblindness have been described in an interview study by Fletcher and Guthrie (2013), who found that persons with deafblindness experience restrictions in daily living and a lack of independence.

In a study of psychological health and its relation to health-related quality of life, Dean et al. (2017) found that, depression, loneliness and lack of social support were associated with poorer psychological wellbeing in persons with USH living in the United Kingdom. Poorer psychological and physical health has been described in Swedish studies that report on the health of persons with USH1, USH2, and USH3, respectively (Wahlqvist et al., 2013, 2016a,b). Compared with a reference group, people with USH2 reported significantly poorer physical and psychological health (Wahlqvist et al., 2013). People with USH2 reported more problems with headache, shoulder and neck pain, feelings of anguish, depression, being under strain, and not being able to concentrate. Suicidal ideation was also severely overrepresented among people with USH2 compared with a reference group. Persons with USH1 reported significantly poorer psychological health and a more exposed situation in terms of social trust and financial situation than did a reference of the Swedish population (Wahlqvist et al., 2016b), major problems with fatigue and loss of confidence were revealed as well as restrictions in social participation due to refraining from going out alone or not receiving help when needed. A study describing the health situation of persons with USH3, stressed the importance of interdisciplinary strategies to facilitate the needs of support for persons with USH3, and deafblindness throughout the life span (Wahlqvist et al., 2016a).

The Bodsworth et al. (2011) study revealed that people with deafblindness received the informal support that they needed from family and friends, and that this help was their major source of support. The participants perceived less formal support than they wanted, and much of the support provided was practical help in terms of activities of daily living. To receive informal and formal support was discussed by participants in a study by Ehn et al. (2019), where the participants when talking about the informal support from families and friends described that it was a support that they could always rely on. However, to get informal support was not always easy in terms of being intrusive or feelings of lost independence. A bio-psychosocial study on deafblindness reported the presence of a fragmented healthcare that was time consuming regarding trying to contact healthcare and welfare, booking interpreters or transportation, and waiting for responses (Möller K., 2003). Lack of knowledge about the consequences of deafblindness was described by Ehn et al. (2019) when describing formal support from professionals. Another study showed that women with USH1 described their contact with low vision clinics and ophthalmology departments as substantially insufficient, uncoordinated and unable to meet their needs (Möller et al., 2009). In a study of older adults, Schneider et al. (2011) reported that support and services were offered based on a single impairment (i.e., hearing or vision) but not the combination of hearing and vision impairment. Bodsworth et al. (2011) stated that out-patient service professionals (i.e., audiologists and optometrists) must be aware of and ask their deafblind patients about symptoms of psychological distress.

The rationale for describing the similarities and differences in health among people with USH is that USH is a rare condition and relevant knowledge is sparse from a bio-psychosocial perspective. From a clinical rehabilitation perspective, it is greatly important to understand the three clinical groups described and whether the similarities and differences found among them are important for rehabilitation. More knowledge within rehabilitation would enable tailored interventions to meet the needs of psychosocial as well as medical concerns.

### Aim

The current study described the similarities and differences among people with USH type 1, 2, or 3 with regard to general health, physical health, psychological health, and social trust as well as financial situation. Furthermore, it explored whether age, gender, clinical diagnosis, visual field, visual acuity, and degree of HI of these participants were associated variables.

## Materials and Methods

### Participants

Adults with USH included in the Swedish Usher Register were asked to participate in a series of studies about their health. In the present study 171 adults with USH living in Sweden are included. The Swedish Usher Register includes participants with USH type 1, 2, or 3. The database is composed of assessments of vision, hearing, balance, and genetics, as well as medical records; it is updated on a regular basis. Medical records were retrieved from the Swedish Usher Register after authorization from the people with USH. People with USH have been recruited to enter their information in the database over the last 30 years; currently, the data of approximately 400 individuals are included in the register. Adults with USH1, 2, or 3 completed two extensive questionnaires about their health: the Health on equal terms (HET) (Public Health Agency of Sweden, 2007) and the Hospital Anxiety and Depression Scale (HAD-scale; Zigmond and Snait, 1983), the Swedish versions of the questionnaires have been used.

All 171 included persons had a clinical diagnosis of USH based on their HI, vision impairment, balance, family histories, and other clinical observations; 90 of these participants had a genetic diagnosis. Data concerning HI, visual impairment (i.e., visual field and visual acuity scores) and genetics are retrieved from the Swedish Usher Register and collected at approximately the same time as the questionnaires. The two questionnaires were sent to 87 persons with USH1, 122 persons with USH2, and 21 persons with USH3. The questionnaires were returned by 60 persons with USH1, 96 persons with USH2, and 15 persons with USH3. However, of these, two persons with USH1, and seven persons with USH2 were excluded because they failed to complete the HAD-scale, see Table 1 for background data.

**TABLE 1:** Background data for persons with USH1, 2 or 3.

	USH1	USH2	USH3
Number	58	89	15
Age years, mean	48	55	41
Age range years	20–78	18–84	19–71
Women	59%	52%	73%
Genetic diagnosis	43%	63%	73%
Degree of hearing impairment, mean (best ear)	99 db	73 db	99 db
Decimal visual acuity, mean (1.0–0.0)	0.5	0.4	0.7
Category of visual field, mean (1–5)^a^	3	4	3

*^*a*^A visual field category of 1 indicates a normal visual field, 5 indicates blindness (Grover et al., 1997).*

### Independent Variables

Analyses were conducted with regard to how different health outcomes varied as a function of numerous factors such as USH category, gender, age, degree of HI, visual field, and visual acuity.

#### USH Category

The three sub-categories of USH are described above. Overall, 58 persons with USH1, 89 persons with USH2, and 15 persons with USH3 were included in the study (see Table 1). The three sub-categories for USH syndrome are equivalent to the clinical diagnosis, and these categories illustrate differences in degree of HI, onset of RP, balance problems, visual field, and visual acuity.

#### Gender and Age

In total, 91 women and 71 men were included in the study. The gender distribution for each group was 24 men and 34 women (USH1), 43 men and 46 women (USH2), and 11 women and 4 men (USH3). The mean overall age was 51 years old, and the age range was 18–84 years old (see Table 1).

#### Hearing

Hearing impairment was assessed. The average pure tone for 0.5, 1, 2, and 4 kHz (PTA4) was used. Thresholds were classified according to the European standard from mild hearing loss to profound hearing loss (hearing levels: mild, over 20 dB and less than 40 dB; moderate, over 40 dB and less than 70 dB; severe, over 70 dB and less than 95 dB; and profound, equal to and over 95 dB) (Stephens, 2001). The degree of HI for the whole USH group was 87 dB, with a range from 40 to 110 dB. Across the three groups, the mean HIs were 99 dB (USH1), 73 dB (USH2), and 99 dB (USH3; see Table 1).

#### Vision

Visual acuity was measured using Snellen chart-based standard test (Grover et al., 1997). The best corrected visual acuity test results were used to evaluate visual acuity function. Decimal visual acuity ranged from 1.00 (perfect visual acuity) to 0.00 (blindness) (Grover et al., 1997). The mean decimal visual acuity of participants was 0.4. Some differences were observed among the USH1, 2, and 3 groups, with mean decimal values of 0.5, 0.4, and 0.7, respectively (see Table 1).

The visual field test (Goldman perimetry) was measured and categorized into five phenotypes (1–5), where 1 denoted normal vision, 2 denoted visual field of partial or complete ring scotoma, 3 denoted a concentric central field loss with remaining peripheral island, 4 denoted a concentric loss and a visual field of ≤10°, and 5 denoted blindness (Grover et al., 1997). The values of the best-corrected visual field measures are presented; the mean for the whole group was 4. The mean visual fields were 3, 4, and 3 for persons with USH1, USH2, and USH3 respectively (see Table 1). In the regression model, decimal visual acuity was dichotomized as 1.00–0.4 (good) and 0.3–0.005 (poor), and visual field was dichotomized as 2–3 (good) or 4–5 (poor).

### Dependent Variables

The Swedish Public Health Agency’s questionnaire HET (Folkhälsomyndigheten, 2007; Public Health Agency of Sweden, 2007) was used, as was the HAD-scale (Zigmond and Snait, 1983).

### The Health on Equal Terms Questionnaire

The HET is a comprehensive questionnaire that contains numerous questions split across the following domains: health, living habits, tobacco and snuff, gambling, alcohol, financial situation, social relationships, and demographics (i.e., gender and age) (Public Health Agency of Sweden, 2007). The present study focused on the questions in the HET that pertained to general health, physical health, psychological health, and social relationships (i.e., social trust and finances). Questions with multiple response alternatives were dichotomized as either “no problem” or “problem.”

### General Health

The HET measures general health via four questions. The first question is “How do you rate your general health?” and is to be answered on a five-point scale ranging from “very good” to “very poor.” Three questions concerning healthy days and activities of daily living were asked (Public Health Agency of Sweden, 2007). The persons estimated how many days that both their physical and psychological health had been affected by poor health over the last 30-day period and whether their physical and psychological poor health had affected their activities of daily living. Having 14 or fewer days of poor physical health, psychological health, or reduced activities of daily living was considered as no problem. Having 15 or more days over the last 30 day-period was considered as a problem (Boström and Nyqvist, 2010).

#### Physical Health

Health on equal terms questions referring to diabetes, asthma, allergy, or high blood pressure were “Have you any of the following diseases?” Answers were given using a four-point scale ranging from “no” to “yes, great distress.” Furthermore, eight questions were related to shoulder and neck pain, back pain/backache/hip pain or ischia, and hand/elbow/leg or knee pains (i.e., pain in the extremities), headache, eczema, incontinence, bowel trouble, and obesity (Public Health Agency of Sweden, 2007). These questions were answered using a three-point scale ranging from “no” to “yes, great discomfort” (Public Health Agency of Sweden, 2007; Boström and Nyqvist, 2010).

#### Psychological Health

Psychological health as assessed by the HET includes questions related to fatigue, sleeping problems, and anxiety/worry or anguish. These questions are answered using a three-point scale from “no,” to “yes, great discomfort.” The psychological health outcomes also included 12 questions regarding abilities over the last few weeks. These questions included topics such as being unable to concentrate, having feelings of worthlessness, and being unable to appreciate the day. Answers were given using a four-point scale ranging from “not at all” to “much more than usual.” A question about stress “Do you feel stressed at present?,” was answered using four-point scale ranging from “not at all” to “very much.” Two questions concerning suicidal behaviors were also included: “Have you at any time found yourself in a situation in which you seriously considered taking your own life?” and “Have you ever tried to take your own life?” Possible answers were “no,” “yes, once” and “yes, several times” (Public Health Agency of Sweden, 2007; Boström and Nyqvist, 2010).

### Social Trust and Financial Situation

Within the HET social relationship domain, questions associated with social trust were asked. These questions were “Do you ever refrain from going out alone for fear of being attacked, robbed or otherwise molested?,” the question could be answered on a three-point scale from “no” to “yes often”; “Have you been subjected to physical violence over the past 12 months?”; and “Have you been subjected to threats of physical violence so that you became frightened over the past 12 months?,” the answer could be “yes” or “no.” Finally, “Have you been treated or received in such a way that you have felt wronged over the past three months?,” the answerer could be given on a three-point scale from “no” to “yes, several times.” Additional questions concerns having someone to share one’s innermost feelings and whom to confide, the possibility to obtaining help if needed and believing that most people can be trusted (Public Health Agency of Sweden, 2007).

The respondents answered questions about their financial situation. The questions were “in case of an unforeseen situation in which you had to get hold of 15,000 Swedish Crowns in a week, could you manage this?” The answer could be “yes” or “no.” The other question asked was “if during the past 12 months that you have had difficulty in managing your expenditure for food, rent, bills and so on?” Answers were on a three-point scale ranging from “no” to “yes, on several occasions” (Public Health Agency of Sweden, 2007).

### The Hospital Anxiety and Depression Scale

The HAD-scale was developed by Zigmond and Snaith in early 80s. It consists of 14 questions, seven of which refer to anxiety or depression. The HAD-scale has been widely used in both clinical settings and among the general population to identify cases of anxiety and depression. Summation indices for each subscale are calculated, and a cut-off for anxiety and depression is set as 7/8 for probable cases (Zigmond and Snait, 1983).

### Statistics

In addition to descriptive statistics, the Kruskal–Wallis test was used to examine the differences among the USH groups with regard to health (i.e., general health, physical health, and psychological health) and social trust/financial situation outcomes. A logistic regression analysis was used to determine whether the independent variables associated with health, social trust and financial situations remained significant after adjustment. Independent variables such as demographics and, clinical diagnosis as well as visual field, visual acuity and HI were used to examine general health, physical health, psychological health, and social trust and financial outcomes). A reference category was determined for each independent variable.

### Ethical Approval

The Ethics Committee of Linköping University Hospital, and the Institutional Review Board of the Boys Town National Research Hospital in Omaha, United States, in 1990 and 1997 approved the use of the material in the Usher Register for research. In 2012, the Ethics Committee of Uppsala approved the translation of the HET and HAD-scale and to send these forms to persons with USH1 to collect data on their health and wellbeing (Dnr 2012/515). All persons with USH signed informed consent documents to participate in the clinical and genetic research on USH.

## Results

The results described the similarities and differences among persons with USH1, 2, or 3 with regard to general health, physical health, psychological health, social trust, and finances. The results also present the analysis of which dependent health outcomes were associated with the controlled independent variables demographics, clinical diagnosis, degree of HI, visual field and visual acuity, and the extent to which the variables affected the health outcome.

### General Health

As Table 2 shows, no differences in self-assessed poor health were observed among persons with USH1, 2, or 3. Group comparisons revealed no significant differences for the frequency of physical poor health days, psychological poor health days or days when the capacity for work and activities of daily living had been lowered. However, a pattern was observed in the differences between the reported frequencies of physical poor days between persons with either USH 1 or USH2 and those with USH3. Persons with USH3 reported fewer poor physical health days.

**TABLE 2:** Percent and χ^2^-test for general health outcomes among persons with USH1, USH2, or USH3.

General Health							
	**%**						
	USH1 *n* = 58	USH2 *n* = 89	USH3 *n* = 15	χ^2^	df	*p*-value	Group Comparison
Poor health	9	10	7	2.23	4	0.69	USH3 vs USH1 vs USH2 ns^c^
Physical health days^a^	27	26	8	2.31	2	0.32	USH3 vs USH2 vs USH1 ns^c^
Psychological health days^a^ ≥15	23	24	23	0.02	2	0.99	USH3, USH1 vs USH2 ns^c^
Prevented capacity for work and ADL^a,b^	22	16	29	1.72	2	0.42	USH2 vs USH1 vs USH3 ns^c^

*^*a*^Having ≥15 days over the last 30-day period was defined as poor health days; alternatively, physical and psychological poor health shows a lowered capacity for work and ADL. ^*b*^Activities of daily living (ADL). ^*c*^ns, indicates non-significant group comparison. Group comparisons were performed with the Kruskal–Wallis test. Significance was set at *p* ≤ 0.05.*

Having 15 or more poor psychological health days was equally reported within the USH1, 2, and 3 groups. Persons with USH2 reported fewer problems with a lowered capacity for work and activities of daily living due to poor physical and psychological health than did those with USH1 or USH3. However, this difference was not significant. Persons with USH3 most frequently reported problems with lowered capacity for work and activities of daily living (Table 2).

The regression model regarding how the independent variables were associated with the general dependent outcomes revealed that age had a significantly high OR (4,07) with regard to the dependent outcome poor health (Table 6). This result shows that the risk of reporting poor general health increased by four times as participants aged.

### Physical Health

Significant differences were observed in three of thirteen physical health outcomes: tinnitus; hand, elbow, knee, and leg pain; bowel problems (Table 3). When in-group comparisons were performed among the USH groups, the significant difference in tinnitus between USH1 and USH2 remained. This difference was not significant for USH3; nevertheless, a large difference remained between persons with USH1 and those with USH3 (Table 3). Persons with USH3 reported more problems with tinnitus than did those with USH1.

**TABLE 3:** Percent and χ^2^-test for physical health outcomes for persons with USH1, USH2, or USH3.

Physical Health							
	%						
	USH1 *n* = 58	USH2 *n* = 89	USH3 *n* = 15	χ^2^	df	*p*-value	Group comparison
Headache	40	48	73	5.44	2	0.07	USH1 vs USH3 sig. USH2 ns^a^
Tinnitus	7	44	40	22.94	2	0.00	USH1 vs USH2 sig. USH3 ns^a^
Pain in shoulders, neck	43	61	67	5.58	2	0.06	USH1 vs USH2 vs USH3 ns^a^
Back pain	45	52	53	0.87	2	0.65	USH1 vs USH2 vs USH3 ns^a^
Pain in hand, elbow, knee, legs	24	40	53	6.28	2	0.04	USH1 vs USH2 vs USH3ns^a^
Eczema, skin rashes	22	29	33	1.06	2	0.59	USH1 vs USH2 vs USH3ns^a^
Incontinence	10	16	7	1.55	2	0.46	USH3 vs USH1 vs USH2 ns^a^
Bowel trouble	19	27	53	7.24	2	0.03	USH3 vs USH1 sig. USH2 ns^a^
Obesity	21	33	27	2.26	2	0.32	USH1 vs USH3 vs USH2 ns^a^
Diabetes	3	6	0	1.37	2	0.50	USH3 vs USH1 vs USH2 ns^a^
Asthma	3	12	13	3.24	2	0.20	USH1 vs USH2, USH3ns^a^
Allergy	21	24	14	0.64	2	0.73	USH3 vs USH1 vs USH2 ns^a^
High blood pressure	7	11	7	0.68	2	0.71	USH3, USH1 vs USH2 ns^a^

*^*a*^ns, indicates non-significant group comparison. Group comparisons were performed with the Kruskal–Wallis test. Significance was set at *p* ≤ 0.05.*

The logistic regression model regarding how different independent variables contributed to poor physical health outcomes did not reveal a clear tendency, where only one variable contributed to poor physical health outcomes. Usher syndrome category or gender was associated with a higher risk for poor outcome on six of the physical outcomes controlled. These findings show that if belonging to a “higher” USH category (i.e., USH2 or USH3) was associated with a higher risk of tinnitus; hand, knee or leg pain; and bowel trouble (table 6). Women were at higher risk for having problems with headache, shoulder and neck pain, and incontinence (Table 6). Older participants had a significant higher risk for diabetes and high blood pressure outcomes. Finally, poor visual field was significantly related to a higher risk of allergy (Table 6).

**TABLE 6:** The significant OR and 95% confidence intervals for the independent variables associated with dependent outcomes^a^.

	Independent variables					
Dependent outcome measures	USH Category	Gender	Age	Visual field	Visual acuity	Hearing impairment
Poor health			4.07 (1.04–15.97)			
Headache		2.56 (1.19–5.51)				
Tinnitus	3.43 (1.59–7.42)					
Pain shoulders. neck		3.06 (1.46–6.45)				
Pain hand. elbow. knee. legs	2.16 (1.10–4.27)					
Incontinence		3.98 (1.126–14.060)				
Bowel trouble	2.66 (1.28–5.51)					
Diabetes			6.12 (1.43–26.21)			
Allergy				3.22 (1.09–9.46)		
High blood pressure			2.90 (1.10–7.63)			
Fatigue	2.65 (1.04–6.77)					
Sleeping problems	2.47 (1.22–5.01)		2.06 (1.22–3.46)			
Lost sleep over worry			2.06 (1.03–4.14)			
Manage problems				0.19 (0.05–2.10)		
Incapable of making decisions					0.10 (0.01–0.75)	
Suicide thoughts						1.77 (1.01–3.11)
Suicide attempts			2.31 (1.06–5.05)			
Refrain from going out alone		6.70 (2.95–15.24)				
No general trust in most people		1.84 (1.08–3.12)			0.31 (0.10–0.97)	
No one to share innermost feelings with and confide in			2.14 (1.14–4.02)		0.18 (0.05–0.71)	
Violated			0.57 (0.33–0.96)			
Difficult financial situation	2.26 (1.07–4.76)					

*^*a*^The table concerns the dependent outcomes where the logistic regression model found a significant OR with regard to any of the controlled independent variables. *p*-Value ≤ 0.05.*

### Psychological Health

Twenty psychological health outcomes were measured, of which two were significant: fatigue and suicidal thoughts (Table 4). The group comparison regarding fatigue revealed that the difference between persons with USH3 and those with USH1 was significant; specifically, persons with USH3 reported far more problems than those with USH1 (93% compared with 62%). The difference among persons with USH2, compared to those with USH1 and USH3 respectively, were not significant. Nevertheless, a pattern was observed such that problems with fatigue increased across clinical type (USH1, USH2, and USH3). The same pattern of significance was revealed for persons with USH3 and those with USH1, but not those with USH2 with regard to suicidal thoughts (Table 4).

**TABLE 4:** Percent and χ^2^-test for psychological health outcomes among persons with USH1, USH2, or USH3.

Psychological Health							
	%						
	USH1 *n* = 58	USH2 *n* = 89	USH3 *n* = 15	χ^2^	df	*p*-value	Group comparison
Worry	36	43	64	3.66	2	0.16	USH3 vs USH2, USH1 ns^b^
Anxiety^a^	26	28	27	0.08	2	0.96	USH2 vs USH3 vs USH1 ns ns^b^
Depression^a^	20	14	20	1.31	2	0.52	USH1, USH3 vs USH2 ns^b^
Fatigue	62	77	93	7.52	2	0.02	USH3 vs USH1 sig. USH2ns^b^
Sleeping problems	26	42	53	5.29	2	0.07	USH3 vs USH2, USH1 ns^b^
Concentration	9	19	13	3.05	2	0.22	USH2 vs USH3, USH1 ns^b^
Appreciate the day	10	11	13	0.11	2	0.95	USH3 vs USH2 vs USH1 ns^b^
Lost sleep over worry	19	9	20	3.56	2	0.17	USH3 vs USH1 vs USH2 ns^b^
Managed problems	11	11	13	0.07	2	0.97	USH3 vs USH2, USH1 ns^b^
Accomplished things	10	18	7	2.45	2	0.29	USH2 vs USH1, USH3 ns^b^
Feeling dejected and depressed	19	20	13	0.40	2	0.82	USH2 vs USH1 vs USH3 ns^b^
Incapable of making decisions	7	8	7	0.05	2	0.98	USH2 vs USH3, USH1 ns^b^
Losing confidence	16	9	7	1.85	2	0.40	USH1 vs USH2 vs USH3 ns^b^
Constantly under strain/tension	26	18	27	1.54	2	0.46	USH3 vs USH1 vs USH2 ns^b^
Feeling worthless	21	14	20	1.45	2	0.49	USH1 vs USH3 vs USH2 ns^b^
Incapable of facing up to problems	18	9	20	3.17	2	0.21	USH3 vs USH1 vs USH2 ns^b^
Unhappy	19	16	13	0.46	2	0.80	USH1 vs USH2, USH3 ns^b^
Stressed	14	8	13	7.87	4	0.10	USH1 vs USH3 vs USH2 ns^b^
Suicidal thoughts	30	23	53	6.23	2	0.04	USH3 vs USH1 vs USH2 ns^b^
Suicide attempts	16	9	20	2.27	2	0.32	USH3 vs USH1 vs USH2 ns^b^

*^*a*^Probable cases of anxiety and depression according to the HAD scale (Zigmond and Snait, 1983). ^*b*^ns, indicates non-significant group comparison. Group comparisons were performed with the Kruskal–Wallis test. Significance was set at *p* ≤ 0.05.*

A non-significant pattern was identified with regard to psychological health outcomes in which persons with USH3 reported the most problems for ten of the outcomes. Persons with USH2 reported the most problems for five outcomes; and persons with USH1reported the most problems for four outcomes (Table 4).

The logistic regression model regarding how different independent variables contributed to poor psychological health outcomes did not reveal a clear predictor of poor psychological outcomes. Of the seven psychological health outcomes included in the model age was associated with a significant OR for three of the outcomes. However, both age and USH category had significant ORs with regard to sleeping problems. Other independent variables were associated with different psychological outcomes (Table 6). Interestingly, the association between visual field and managing problems was negative (B −1,67, SE 0,71, Wald 5,54, df 1, *p*-value 0.02, Exp B 0.19, CI 0.05–2.10); thus, those who had better visual fields were at a higher risk for poor problem management. A negative association was found between visual acuity and being incapable of making decisions (B −2,34, SE 1.04, Wald 5.027, df 1, *p*-value 0.025, Exp B 0.10, CI 0.01–0.75).

### Social Trust and Financial Situation

Six of nine social trust and financial situation outcomes presented with significant differences (Table 5). Regardless of significance, persons with USH3 reported more problems (5/9 outcomes) closely followed by those with USH1 (4/9 outcomes). Persons with USH2 reported the fewest problems except with regard to difficult financial situations; here, persons with USH1 reported the fewest problems. Group comparisons revealed significant differences among all three USH categories regarding violence and difficult financial situations (Table 5).

**TABLE 5:** Percent and χ^2^-test for social trust and financial situation outcomes among persons with USH1, USH2, or USH3.

Social Trust							
	%						
	USH1 *n* = 58	USH2 *n* = 89	USH3 *n* = 15	χ^2^	df	*p*-value	Group comparison
Refrain from going out alone	59	41	67	6.10	2	0.05	USH3 vs USH1, USH2 ns^a^
Not seeking help when needed	26	5	7	15.71	2	0.00	USH2 vs USH1 sig, USH3 ns^a^
No general trust in most people	43	28	53	5.19	2	0.08	USH3 vs USH1, USH2 ns^a^
No one to share innermost feelings with and confide in	39	12	13	14.66	2	0.00	USH1 vs USH2 sig, USH3 ns^a^
Feeling violated	28	27	53	4.46	2	0.11	USH3 vs USH2 sig, USH1 ns^a^
Feeling threatened	9	2	7	3.35	2	0.19	USH1, USH3, USH2 ns^a^
Experienced Violence	2	1	13	8.09	2	0.02	USH3 vs USH1, USH2 sig

Financial Situation							

Difficult financial situation	16	19	47	7.26	2	0.03	USH3 vs USH2, USH1 sig
Unforeseen situation	43	24	40	6.13	2	0.05	USH1, USH3 vs USH2 ns^a^

*^*a*^ns, indicates non-significant group comparison. Group comparisons were performed with the Kruskal-Wallis test. Significance was set at *p* ≤ 0.05.*

The regression model performed for the social trust and financial situation outcomes did not reveal a significant predictor of poor outcomes (Table 6). Different independent measures contributed for each social trust and financial situation outcome. Gender/visual acuity and age/visual acuity significantly and negatively predicted general trust in most people and no one to share innermost feelings and whom to confide, respectively (B −1.19, SE 0.59, Wald 4.06, df 1, p-value 0.04, Exp B 0.31, CI 0.10–0.97; B −1.71, SE 0.70, Wald 6.01, df 1, p-value 0.01, Exp B 0.18, CI 0.05–0.71; see Table 6). This finding implies that persons with USH and better visual acuity are at higher risk for having a lack of general trust and not having someone with whom to share their innermost feelings and in whom to confide. The same pattern was revealed between being violated and age (B −0.57, SE 0.27, Wald 4.45, df 1, *p*-value 0.04, Exp B 0.57, CI 0.33–0.96; Table 6).

## Discussion

Both similarities and differences were identified among the USH groups with regard to general health, physical health, psychological health, social trust, and financial situation. Persons with USH3 tended to have poor outcomes such as headaches; hand, knee, leg, and elbow pain; fatigue; sleeping problems; and suicidal thoughts. These participants also tended to refrain from going out and did not trust most people in general. Persons with USH1 presented with a shifting of problems, primarily concerning psychological health and problems with social trust and finances. Persons with USH2 had problems that were more evenly distributed across physical health, psychological health, and social trust (see Tables 2–5). Likewise, the regression model showed high ORs for USH category, indicating that the USH3 group was at higher risk (Table 6). However, because few persons had USH3 (*n* = 15) these results should be interpreted with caution, and more research (both longitudinal and qualitative) are needed to examine the variables that contribute to the complexity of living with a progressive condition such as USH further.

The current results are ambiguous as to which variables contributed the most to the poor health, social trust and financial outcomes; each outcome was associated with a different predictor (Table 6). These findings do not mean that the independent variables lack importance; rather, they indicate the complexity of living with a progressive degenerative syndrome such as USH. Differences in background variables such as gender, age, visual acuity, visual field and degree of HI were observed (Table 1), and these variables were associated with high ORs for certain outcomes (Table 6). For example, one OR showed that women have a higher risk of reporting headache (Table 6). Furthermore, a higher age was related to the risks for diabetes, high blood pressure, sleeping problems, lost sleep over worry, and suicide attempts. Another OR revealed a negative relationship between risk of violation and age (Table 6), such that younger persons have a higher risk of being violated.

Deafblindness such as that found in USH is a complex phenomenon. We empirically examined numerous independent variables to determine how they are associated with different health outcomes. The results did not reveal connections in terms of linear causality; thus, the risk of a poor health, social and finance outcome depended on the independent variable. Concluding that persons with USH automatically have poor health outcomes or more problems with social trust or finances as their vision deteriorates is not possible based on the results of the present study. The same reasoning can be made with regard to the degree of HI. However, vision and hearing are highly complementary senses (Möller C., 2003) used to communicate and interact with others; when these senses deteriorate, individual’s social lives might become restricted (Fletcher and Guthrie, 2013), thereby affecting their health and social trust (Wahlqvist et al., 2013).

This study and previous studies both from the current research group (Wahlqvist et al., 2013, 2016a, 2016b) and others (see for example Miner, 1995, 1997; Schneider, 2006; Bodsworth et al., 2011; Ellis and Hodges, 2013; Dean et al., 2017) have found that persons with USH or deafblindness feel exposed with regard to poor physical health, psychological health and social withdrawal. However, the mechanisms associated with the health of people with USH their lack of social trust and financial problems are not clear. Social support has been described as a variable that affects the psychological health for persons with USH positively (Dean et al., 2017). To have strategies to concur difficulties when they occur (Ehn et al., 2019) has also been described as positive as well as remaining active through work (Ehn et al., 2016, 2018) or by other engagements (Ehn et al., 2019). Still, variables affecting the health of people with USH need to be studied from a life course, bio-psychosocial perspective to reveal possible interaction effects and consequences (Arcous et al., 2019).

Deafblindness such as that observed in USH is described as a disability that affects communication, giving and receiving information and orientation (Fletcher and Guthrie, 2013). Hearing and vision are greatly involved when we communicate with others and play roles in obtaining information and orientation. As vision deteriorates, evident for all persons with USH regardless of clinical type, and for those with USH 3 the deterioration also affects hearing, the mode of communication and interaction with others and the environment comes under attack. This effect might have implications for the possibility of living an independent life as has been described in previous research (Ellis and Hodges, 2013; Fletcher and Guthrie, 2013; Hersh, 2013). Communication barriers (which concern a lack of knowledge from others), negative attitudes and misconceptions regarding of what others think a person with deafblindness is capable of have been reported (Schneider, 2006; Hersh, 2013). The present study did not examine the communication strategies that the persons with USH use. However, some of the problems reported might concern a compromised communication situation. The efforts made to see, hear and follow what is being said in a conversation or what is happening in the environment might lead to fatigue, strain and shoulder and neck pain.

Because USH is a rare syndrome, healthcare and rehabilitation setting professionals seldom come in contact with people with USH, and previous research has found that the healthcare and support that is offered does not meet the needs of the people with deafblindness or USH because it is fragmented, lacks coordination and is time consuming (Bodsworth et al., 2011; Möller K., 2003; Möller et al., 2009; Schneider et al., 2011). Therefore, it is of great importance to gain knowledge regarding how one’s physical, psychological and social health affects people with USH in their daily lives from a life course perspective; this goal requires an interdisciplinary bio-psychosocial perspective. If unknown, then support and rehabilitation efforts might have limited effects. People with USH also report, spending much effort in explaining their life situation to professionals and others (Ehn et al., 2019).

### Implications for Rehabilitation

During rehabilitation or in other healthcare settings, the whole person must be taken into account; hence, a bio-psychosocial perspective is necessary to understand the general, physical, psychological, and social health of persons with USH. To focus on a single impairment (i.e., hearing or vision) in the rehabilitation or healthcare setting reduces a complex living situation and does not consider deafblindness as a distinct disability with special consequences for the individual or the adjustments that must be made by society.

## Conclusion

None of the independent variables examined in the current study fully explain the poor health of persons with USH. The independent variables that contributed the most to poor health outcomes were ambiguous. Additional research is needed to examine the consequences for health and other variables or mechanisms regarding persons with USH. An evidence-based rehabilitation setting that provides help and support for people with USH should rely on the individual’s knowledge about their life situation and challenges, research and professional knowledge. The observations of the associations between the independent variables and poor health, social trust and finances made in the present study are important to bear in mind in a rehabilitation setting; however, they, do not fully explain how people with USH actually feel or rate their health. More research is needed to confirm the knowledge that exists within the clinical setting and the life stories told by the people with USH to merge existing knowledge into a rehabilitation setting based on evidence.

## Data Availability Statement

Due to privacy concerns, the data will not be made publicly available.

## Ethics Statement

The studies involving human participants were reviewed and approved by the Ethics Committee of Linköping University Hospital, and the Institutional Review Board of the Boys Town National Research Hospital in Omaha, United States, in 1990 and 1997 approved the use of the material in the Usher Register for research. The Ethics Committee of Uppsala approved the translation of the HET and HAD-scale and to send these forms to persons with USH1 to collect data on their health and wellbeing (Dnr 2012/515). The patients/participants provided their written informed consent to participate in this study.

## Author Contributions

MW was responsible for the data collection, statistical analysis, and writing. CM, BD, and KM contributed for the discussions of analysis, results, and writing. All authors have contributed to the design, analysis, and writing of the manuscript and the study.

## Conflict of Interest

The authors declare that the research was conducted in the absence of any commercial or financial relationships that could be construed as a potential conflict of interest.
